# A Feedback Inhibition between miRNA-127 and TGFβ/c-Jun Cascade in HCC Cell Migration via MMP13

**DOI:** 10.1371/journal.pone.0065256

**Published:** 2013-06-07

**Authors:** Zhihong Yang, Yuxia Zhang, Li Wang

**Affiliations:** Departments of Medicine and Oncological Sciences, Huntsman Cancer Institute, University of Utah School of Medicine, Salt Lake City, Utah, United States of America; University of Hong Kong, Hong Kong

## Abstract

Hepatocellular carcinoma (HCC) is the fifth most common cancer worldwide and is increasing in frequency in the U.S. The major reason for the low postoperative survival rate of HCC is widespread intrahepatic metastasis or invasion, and activation of TGFβ signaling is associated with the invasive phenotype. This study aims at determining the novel function of miR-127 in modulating HCC migration. Overexpression of miR-127 inhibits HCC cell migration, invasion and tumor growth in nude mice. MiR-127 directly represses matrix metalloproteinase 13 (MMP13) 3′UTR activity and protein expression, and diminishes MMP13/TGFβ-induced HCC migration. In turn, TGFβ decreases miR-127 expression by enhancing c-Jun-mediated inhibition of miR-127 promoter activity. In contrast, p53 transactivates miR-127 promoter and induces miR-127 expression, which is antagonized by c-Jun. The inhibition of miR-127 by c-Jun is through TGFβ-mediated ERK and JNK pathways. The lower miR-127 expression shows a negative correlation with the higher MMP13 expression in a subset of human HCC specimens. This is the first report elucidating a feedback regulation between miR-127 and the TGFβ/c-Jun cascade in HCC migration via MMP13 that involves a crosstalk between the oncogene c-Jun and tumor suppressor p53.

## Introduction

The microRNA-127 (miR-127) is located within the imprinted Dlk1/Gtl2 region expressed from the maternal chromosome [1]. MiR-127 is clustered with miR-433 and both miRNA primary transcripts are controlled by nuclear receptor signaling involving small heterodimer partner (SHP) and estrogen related receptor gamma (ERRγ) [2]–[4]. Thus far, the *in vivo* physiological function of miR-127 has not been studied and remains largely unknown. In particular, conflicting information is available regarding the potential role of miR-127 in human cancers.

The first report suggesting that miR-127 is a putative tumor suppressor was based on observations that miR-127 levels were induced by the chromatin-modifying drug 5′-aza-2′-deoxycytidine (Aza) in a panel of human cancer cell lines including T24, HCT116, Hela, NCCIT, and Ramos cells [5]. However, miRNA expression profiling in early stage invasive squamous cell carcinomas (ISCC) revealed a significant correlation between the increased expression of miR-127 and lymph mode metastasis [6]. In addition, miR-127 was part of the miRNA signature that was upregulated in acute myeloid leukaemia (AML) [7] and in nodal diffuse large B-cell lymphomas [8]. In human breast cancer [9] and in a rat model of liver carcinogenesis induced by a methyl-deficient diet [10], the levels of miR-127 were decreased. A recent report showed that upregulation of miR-127-3p was associated with KRAS mutation in colorectal cancer [11]. Despite these existing literature reports pointing to a role for miR-127 in various cancers, no detailed functional and mechanistic studies have been done.

This study was undertaken to characterize the function of miR-127 in human hepatocellular carcinoma (HCC). We show that overexpression of miR-127 represses HCC migration, invasion, and tumor growth. The effect of miR-127 is through direct inhibition of TGFβ-mediated activation of matrix metallopeptidase 13 (MMP13, also known as collagenase-3). Interestingly, TGFβ inhibits miR-127 transcription through activation of c-Jun via extracellular signal-regulated kinases (ERK) and the c-Jun amino-terminal kinases (JNK) pathways, which counteracts the stimulation of miR-127 by p53. We further show that the activation of TGFβ signature in a subset of HCC is at least in part attributed to the downregulation of miR-127. Our results demonstrate for the first time that miR-127 serves as a potential tumor suppressor in HCC by antagonizing TGFβ-mediated HCC cell migration. The results are important because it is the first step in a continuum of research that is expected to facilitate the development of miRNA based therapeutic strategies that will enable TGFβ-targeted treatment of HCC.

## Materials and Methods

### Plasmids and Reagents

The human MMP13 3′UTR and PPP1R9B 3′UTR were cloned into pMIR-Reporter (Invitrogen) using primers MMP13-3′UTR forward (5′-TCAGACTAGTATCCTGAAGAGCATTTGG-3′) and backward (5′-TGACGAGCTCCAACAGTGTCTCTGAGCA-3′), and PPP1R9B-3′UTR forward (5′-GGGGTACCCAGGAATCATTCCATGAC-3′) and backward (5′-CCGCTCGAGCTCTGGGGGCTGCTTTTA-3′). Mutations in pMIR-Reporter-MMP13-3′UTR were generated with a SiteMutant Kit using primers MMP13-3′UTR mut 114 forward (5′-AGGAGAAAGCTTGGTTCTGTGAACAA CGATCAGTAAGTTATCTTTGAATATGTA-3′) and backward (5′-TACATATTCAAAGAT AACTTACTGATCGTTGTTCACAGAACCAAGCTTTCTCCTG-3′). The plasmids were confirmed by sequencing. The miR-127 expression plasmid pTarget-miR-127 was generated as described [3]. pSicoGFP control and pSico-miR-127GFP vectors were generously provided by Dr. Dianhua Jiang (Duke University) [12]. Recombinant adenoviral stocks expressing Cre recombinase (Ad-cre) were purchased from the Gene Transfer Vector Core at the University of Iowa. TGFβ and MMP13 siRNA were purchased from Sigma. NF-κB inhibitor (BAY-11-7082), JNK inhibitor (SP 600125), ERK1/2 inhibitor (PD 98059), p38 inhibitor (SB 203580), and TGFβ receptor inhibitor (SB 431542) were purchased from Cayman. Recombinant MMP13 protein was purchased from ProSpec-Tany TechnoGene Ltd. MMP13 inhibitor was purchased from Santa Cruz. Matrigel was purchased from BD Biosciences. MHCC97H and MHCC97L cells [13] were a gift from Dr. Pingyi Xu (Sun Yat-sen University, Guangzhou, China). HepG2, Hepa-1, Hep3B, and Huh7 cells have been described [14].

### Generation of miR-127 Expressing Cells

The cells were generated following a published method [12]. We first established a MHCC97H-Luc (97HLuc) cell line in which a constitutive luciferase reporter was stably expressed using a retrovirus containing pLuc-puro [14]. PsicoGFP and Psico-miR-127GFP plasmids were transfected into 293T cells by the calcium phosphate method to produce viral particles. Lentiviral supernatants were collected 48 hr after transfection, passed through a 0.22-mm filter, and used to infect 97HLuc cells, which generated 97HLuc-SicoGFP and 97HLuc-miR-127GFP cells, respectively. Ad-cre virus (100 PFU) was then used to infect 97HLuc-SicoGFP or 97HLuc-miR-127GFP cells, which removed the GFP marker and generated 97HLuc-Sico and 97HLuc-miR-127 cells, respectively. The 97HLuc-Sico and 97HLuc-miR-127 cells were used in Xenograft mouse model.

### Xenograft Mouse Model

Six-week-old female athymic nude mice nu/nu were used for MHCC97H tumor xenografts, as described previously [14], [15]. Both flanks of each mouse were injected with 0.5×10^6^ 97HLuc-Sico or 97HLuc-miR-127 cells mixed with Matrigel (Invitrogen) in a total volume of 100 µl. Tumor growth was imaged with the Xenogen bioluminescent imaging system. Protocols for animal use were approved by the Institutional Animal Care and use Committee at the University of Utah.

### Wound Healing Assay

The method was described previously [16]. Briefly, MHCC97H cells (1×10^6^, 6-cm plate) were cultured overnight and transfected with pTarget or pTarget-miR-127 the next day using Lipofactamine 2000 (Invitrogen). After 36 hr, cells were grown to confluence and wounded by dragging a 200 µl pipette tip through the monolayer. Cells were washed using pre-warmed PBS to remove cellular debris and allowed to migrate for 16 hr. Wound closure or cell migration images were photographed when the scrape wound was introduced (0 hr) and at a designated time (16 hr) after wounding, using an a DMI6000 inverted microscope. The relative surface traveled by the leading edge was assessed by using LAS AF 6000 1.8.0 software. Three replicates per experiment were performed. Apoptosis and cell proliferation assays were described previously [14], [17].

### Cell Migration and Invasion Assays

The cell migration assays were described previously [18]. Briefly, MHCC97H cells were transfected with the indicated plasmids or anti-microRNAs (Qiagen) for 6 hr. The cells were then serum starved for 24 hr and 5×10^4^ cells were seeded on Transwell inserts (8 µm pore size; Culterx 96 well cell migration assay, cat# 3465-095-K). Cells were allowed to migrate for 16 hr, and the non-migratory cells were removed from the insert with a cotton swab. The migrated cells were fixed for 10 min (3.7% v/v formaldehyde in PBS) before staining with 0.1% crystal violet for 15 min, followed by washing with PBS. Pictures were taken with Microfire/Qcam CCD Olympus 1×81 microscope. Crystal violet stained cells were counted and then lysed with 1% SDS for 30 min and absorbance was measured at 595 nm. Cell invasion assays were performed similarly as migration assays, except that cells were seeded on pre-coated inserts with BME solution (Cultrex 96 well BME cell invasion Assay, cat# 3455-096-K). Invaded cells were treated with Cell Dissociation Solution/Calcein-AM for 1 hr, and fluorescence was measured at 485 nm excitation and 520 nm emission.

### MMP13 ELISA Assay

MHCC97H cells were transfected with the indicated plasmids for 24 hr and treated with agonist for another 24 hr. The levels of secreted MMP13 (active form) in the culture supernatants were measured using Human MMP13 Platinum ELISA kit (eBioscience) following the manufacturer’s instructions.

### Analysis of miRNA Expression

MHCC97H cells were grown to 60–70% confluence and treated with agonist for 24 hr. Cells treated with ethanol served as a negative control. Total RNAs including miRNAs were isolated from cells using a miRNeasy mini kit (Qiagen) and reverse transcribed using a miRNA reverse transcription kit (Qiagen). The miR-127 levels (mature form) were quantified by qPCR using miRNA primer assays Kit (Qiagen). All the kits were used according to the manufacturer’s instructions. U6 transcript was used as an internal control to normalize RNA input.

### Transient Transfection and Luciferase Assay

In brief, cells were transfected with the plasmids as indicated in the Figure legends. Transfection was carried out using Lipofectamine 2000 (Invitrogen). Luciferase activities were measured and normalized against Renilla activities (Promega). Experiments were done in three independent triplicate transfection assays. Detailed methods were described previously [19].

### Statistical Analysis

All the experiments were repeated at least three times, and the error bars represent the standard error of the mean (SEM). Statistical analyses were carried out using Student’s unpaired t test; p<0.01 was considered statistically significant.

## Results

### miR-127 Inhibits HCC Cell Migration and Invasion

**Figure 1:**
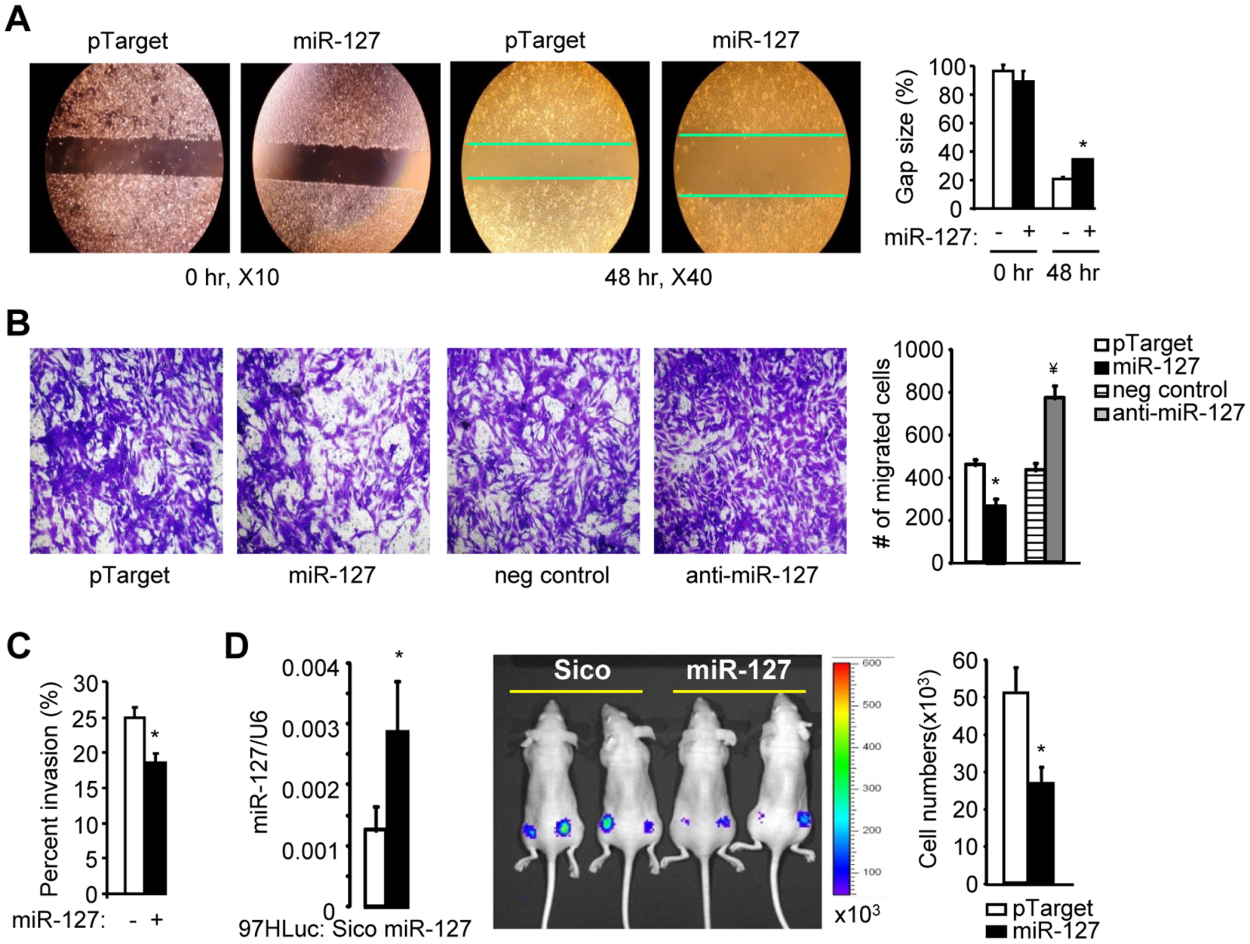
Overexpression of miR-127 inhibits HCC cell migration and tumor growth. (*A*) Wound healing assay. MHCC97H cells were transfected with either an empty vector pTarget (-) or a miR-127 expression vector in pTarget (+) (2 µg). Wound closure was photographed 48 hr later (left) and the residual gap between the migrating cells was quantified (right), which is expressed as a percentage of the initial scraped area. (*B*) Cell migration assay. MHCC97H cells were transfected with pTarget or pTarget-miR127 (2 µg), a negative (neg) control or miR-127 inhibitor (anti-miR-127) (20 nM). Cells that had migrated through the membrane of Transwell inserts were fixed, stained with crystal violet, and visualized by microscopy. (*C*) Cell invasion assay. MHCC97H cells were transfected with pTarget or pTarget-miR127 (2 µg), and seeded onto pre-coated inserts with BME solution and incubated for 16 hr. The invaded cells were dissolved in Cell Dissociation Solution/Calcein-AM and absorbance was measured. (*D*) Tumorigenesis assay. Control 97HLuc-Sico or 97HLuc-miR-127 cells were grafted subcutaneously in the dorsa of athymic mice, and tumor growth was monitored using the Xenogen bioluminescent imaging system by day 9. The number represents tumor images detected (photons/s/cm^2^/steridian). *p<0.01, miR-127 *vs.* pTarget control; ^¥^p<0.01, anti-miR-127 *vs.* negative control.

We conducted several *in vitro* assays to assess the functional role of miR-127 in liver cancer. MHCC97H is a metastatic liver cancer cell line [13], which shows a much greater invasion property than other HCC cells, including HepG2, Hep3B, Huh7, and MHCC97L (pilot study, not shown). The major focus is to determine the inhibitory effect of miR-127 in HCC cell migration and/or invasion, thus a higher invasive cell line, i.e. MHCC97H, was chosen to be the most appropriate cell model for our study. Overexpression of miR-127 decreased the migration rate of MHCC97H by about 40% as examined by a wound healing assay (Fig. 1A), as well as by an alternate Transwell cell migration assay (Fig. 1B, left two). In contrast, knockdown of endogenous miR-127 using a specific miR-127 inhibitor (anti-miR-127) facilitated MHCC97H cell migration (Fig. 1B, right two). The invasive potential of MHCC97H cells was similarly diminished by miR-127 expression as determined by a Transwell cell invasion assay (Fig. 1C). Therefore, migration assays were used in the subsequent experiments throughout the study.

Next, we established control and miR-127 overexpressing cells that also express a constitutive luciferase reporter (see M&M section), which are designated as 97HLuc-Sico (control) and 97HLuc-miR-127 (Fig. 1D, left). qPCR confirmed that miR-127 was ∼2-fold increased in 97HLuc-miR-127 cells, which was consistent with the published literature that used the same miR-127 expression system [12]. Both 97HLuc-Sico and 97HLuc-miR-127 cells were implanted subcutaneously in nude mice and tumor growth was monitored using the Xenogen bioluminescent imaging system [14]. Ectopic expression of miR-127 in 97HLuc-miR-127 cells decreased tumor growth compared with the 97HLuc-Sico cells by day 9 (Fig. 1D, right). The results suggest that miR-127 is likely to function as an inhibitor of HCC.

### miR-127 Decreases MMP13 3′UTR Reporter Activity and Protein Expression

**Figure 2:**
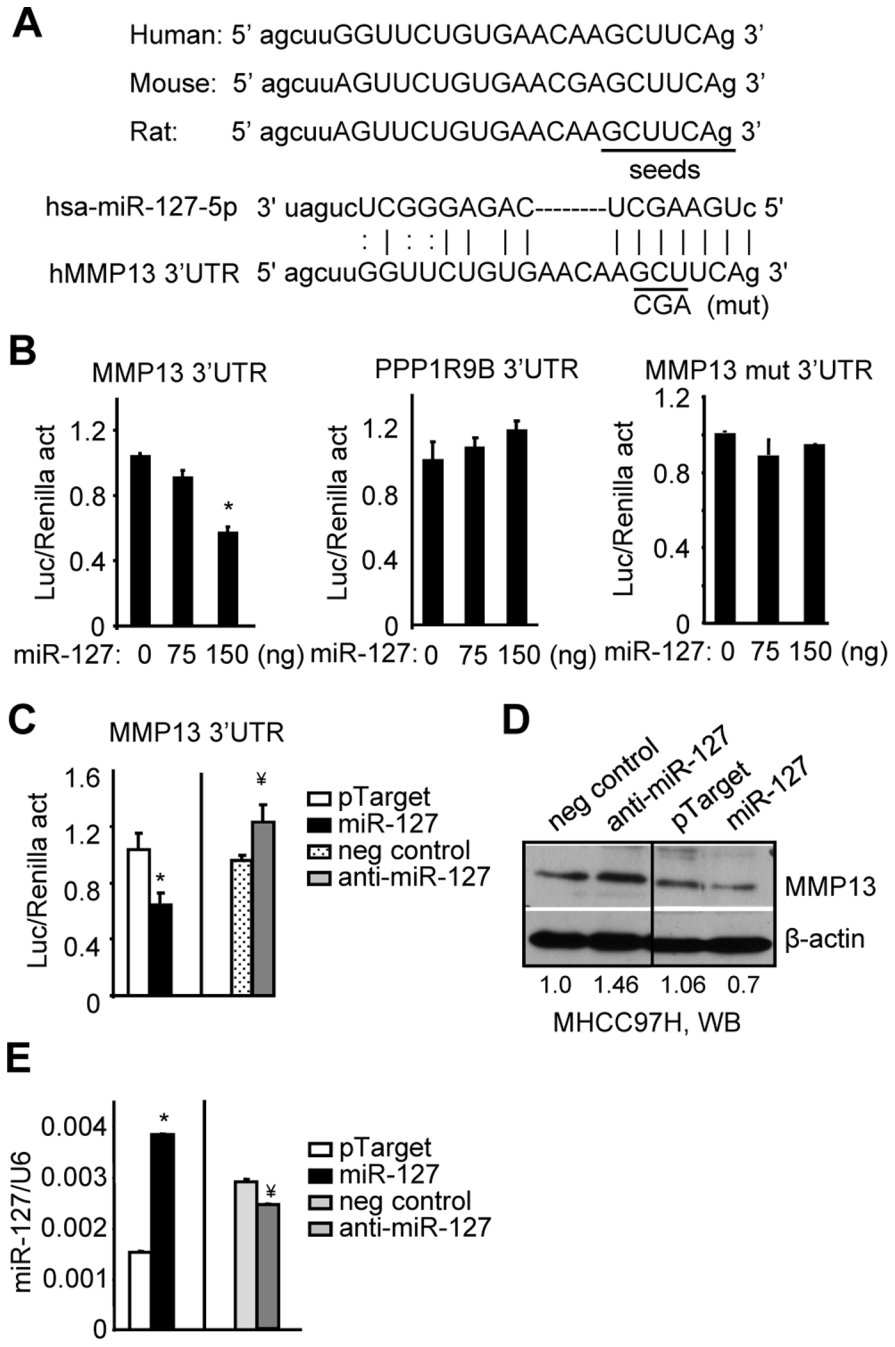
Overexpression of miR-127 inhibits MMP13 3′UTR activity and gene expression. (*A*) Sequence alignment between miR-127 and the 3′UTR of MMP13 in human, mouse, and rat. Solid line: seed match region. (*B*) Transient transfection assay. Hela cells were co-transfected with the control pTarget (0), pTarget-miR-127, and MMP13 (left), PPP1R9B (middle), or MMP13 mutant (mut) 3′UTRs (right). Luciferase (Luc) activity was normalized to Renilla activity (act). PPP1R9B served as a negative control. (*C*) Transient transfection assay to determine the effect of pTarget-miR-127 (100 ng) and miR-127 inhibitor (20 nM) on MMP13 3′UTR reporter activity. (*D*) Western blots (WB) of MMP13 protein expression in MHCC97H cells that were transfected with pTarget-miR127 (2 µg) or treated with a miR-127 inhibitor (anti-miR-127) (20 nM). (**E**) qPCR analysis of miR-127 expression under the same experimental conditions as (*D*). (*B-E):* Data are expressed as means ± SD of triplicate assays. *p<0.01, miR-127 *vs.* pTarget control; ^¥^p<0.01, anti-miR-127 *vs.* negative control.

Two algorithms, TargetScan and miRanda, were used to predict miR-127 target genes that are involved in regulating cell migration/invasion. The human, mouse and rat MMP13 3′UTR shared the same seed region for miR-127 (Fig. 2A), thus MMP13 was predicated to be a target of miR-127. We cloned the human MMP13 3′UTR into a luciferase reporter. Transient transfection assays showed that the MMP13 3′UTR reporter activity was decreased in a dose-dependent fashion by ectopic expression of miR-127 (Fig. 2B, left). On the other hand, PPP1R9B (protein phosphatase 1, regulatory subunit 9B) was also a predicted target of miR-127, but its 3′UTR reporter activity was not altered by miR-127 expression (Fig. 2B, middle). When the miR-127 seed region in the MMP13 3′UTR was mutated, the suppression of MMP13 3′UTR activity by miR-127 was relieved (Fig. 2B, right).

Consistent with these results, inhibition of endogenous miR-127 expression by a specific anti-miR-127 increased MMP13 3′UTR activity (Fig. 2C). In spite of relatively low efficiency of anti-miR-127, the abundance of MMP13 protein in whole cell lysate was increased by anti-miR-127 and reduced by miR-127 overexpression (Fig. 2D). In addition, MMP13 mRNA showed similar changes as its protein upon inhibition or overexpression of miR-127 (not shown), which was in agreement with the inference that a miRNA also represses its target gene mRNA [16]. Overall, the results demonstrate that MMP13 is a direct target gene of miR-127.

### miR-127 Represses HCC Cell Migration Induced by MMP13

**Figure 3:**
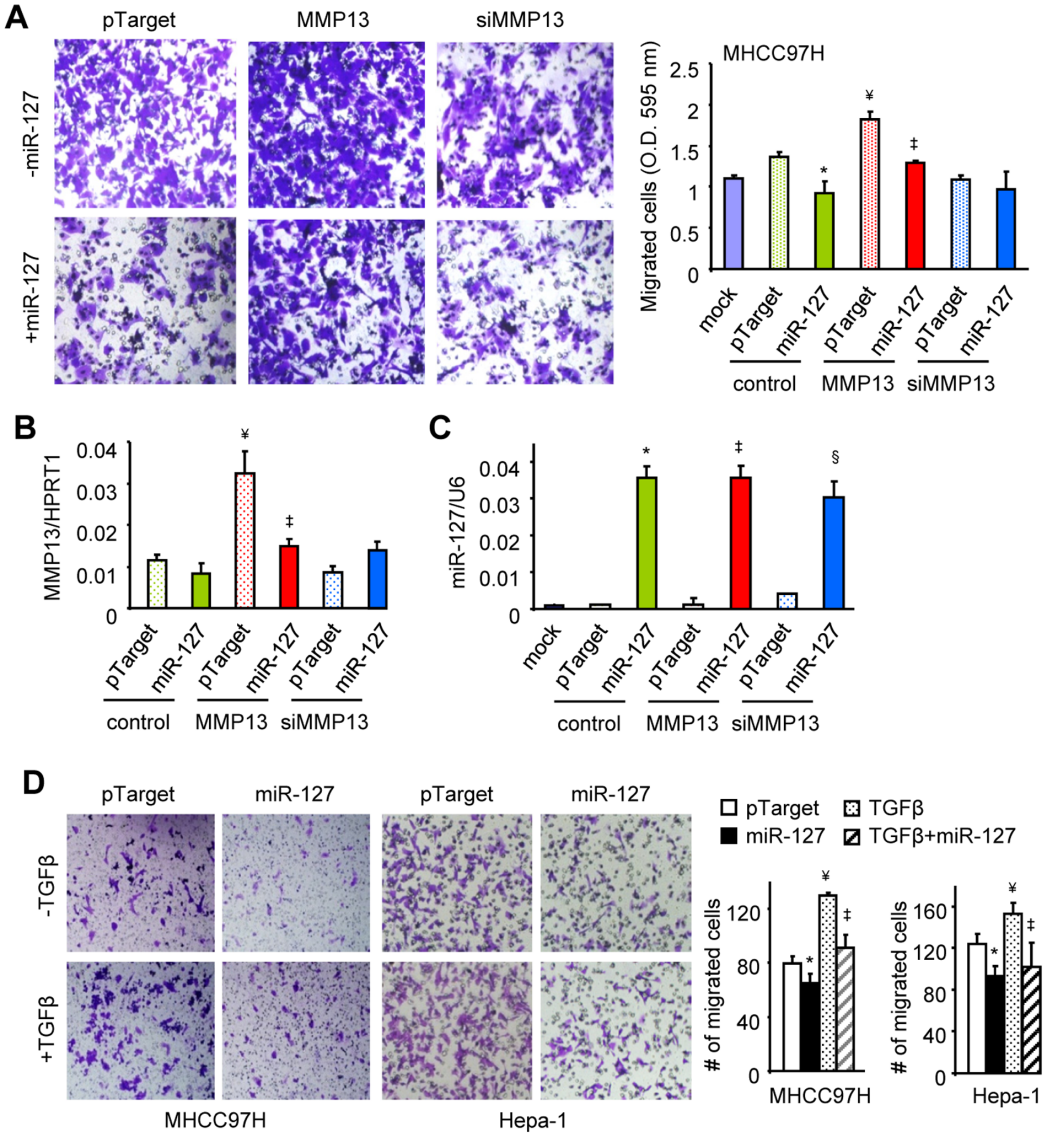
Overexpression of miR-127 inhibits MMP13 and TGFβ-mediated induction of HCC cell migration. (*A*) Cell migration assay. MHCC97H cells were transfected with MMP13 siRNA (siMMP13) (20 nM) in the absence or presence of pTarget or pTarget-miR-127 (2 µg). After cells were seeded onto the inserts, lower chamber media were treated with MMP13 peptide (50 ng/ml) as indicated. Cells that had migrated through the membrane were fixed and stained with crystal violet (left). Quantitative results on the right. (*B-C*) qPCR analysis of MMP13 (*B*) and miR-127 (*C*) expression under the same experimental conditions as (*A*). (*D*) Cell migration assay. MHCC97H and Hepa-1 cells were transfected with pTarget or pTarget-miR-127 (2 µg), in the absence or presence of TGFβ (5 ng/ml). Migrated cells were stained with crystal violet and visualized by microscopy (left). Quantitative results on the right. (*A-D*): *p<0.01, miR-127 *vs.* pTarget in control group; ^¥^p<0.01, pTarget in MMP13 group *vs.* pTarget in control group;^ ‡^p<0.01, miR-127 *vs.* pTarget in MMP13 group; ^§^p<0.01, miR-127 *vs.* pTarget in siMMP13 group.

Upregulation of MMP13 was linked to lymph node metastasis in HCC, suggesting that MMP13 likely regulates liver cancer metastasis [20]. We next examined the effects of miR-127 and MMP13 on HCC cell migration. Treatment of MHCC97H cells with a recombinant human MMP13 protein markedly increased HCC cell migration, which was significantly attenuated by miR-127 overexpression (Fig. 3A). In contrast, knockdown of the endogenous MMP13 using MMP13 specific siRNAs (siMMP13) decreased cell migration. The moderate effect of siMMP13 may be attributed by its low knockdown efficiency (Fig. 3B). The levels of miR-127 were not affected by MMP13 (Fig. 3C). Overall, the results suggest that miR-127 directly counteracts the effect of MMP13 on HCC cell migration.

#### miR-127 attenuates TGFβ-mediated induction of HCC cell migration

Previous studies showed that MMP13 was induced by TGFβ in squamous carcinoma cells [21] and in primary human gingival epithelial cells [22]. Blocking TGFβ signaling reduced migration and invasion of HCC cells [23]. We therefore determined the interaction between miR-127 and TGFβ in controlling HCC cell migration. As expected, TGFβ treatment induced MHCC97H migration and its effect was markedly blunted by co-expression of miR-127 (Fig. 3D). Similar results were observed in mouse HCC Hepa-1 cells. The results provide evidence for miR-127 inhibition of TGFβ-mediated HCC migration.

### TGFβ Enhances AP1-mediated Suppression of miR-127 Expression

**Figure 4:**
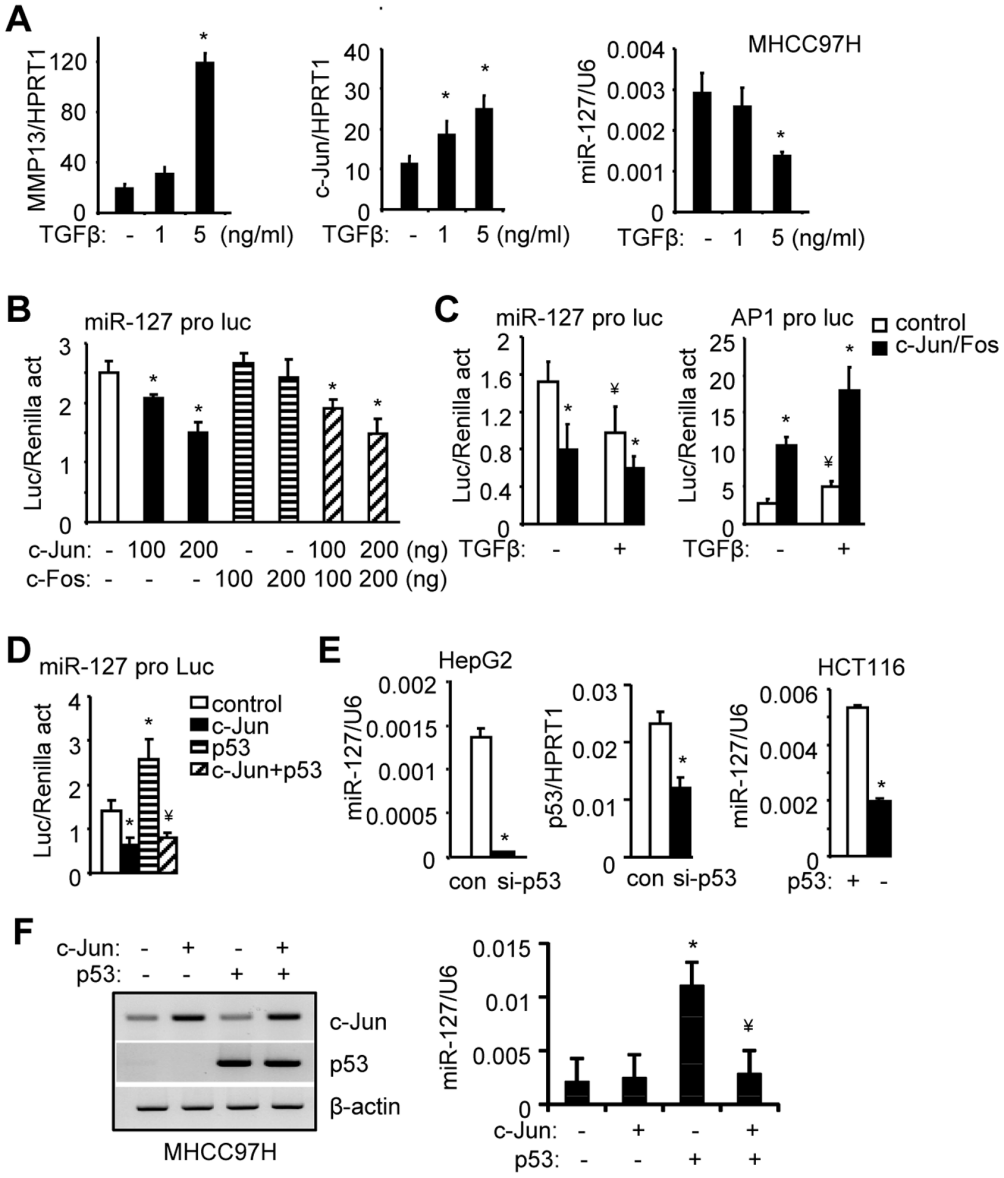
TGFβ represses miR-127 expression by enhancing c-Jun activity. (*A*) qPCR analysis of MMP13 (left), c-Jun (middle), and miR-127 (right) expression in MHCC97H cells treated with TGFβ. *p<0.01, TGFβ (+) *vs.* TGFβ (-) group. (*B*) Transient transfection assay. Hela cells were co-transfected with miR-127 promoter (pro) luciferase (luc) reporter, and c-Jun or c-Fos expression plasmids. *p<0.01, c-Jun (+) *vs.* c-Jun (-) group. (*C)* Transient transfection assay. Hela cells were co-transfected with miR-127 or AP1 promoter reporter along with c-Jun/c-Fos plasmid (100 ng) in the absence or presence of TGFβ (5 ng/ml). *p<0.01, c-Jun/Fos *vs.* control without (-) or with (+) TGFβ; ^¥^p<0.01, control with (+) TGFβ *vs.* control without (-) TGFβ. (*D*) Transient transfection assay. Hela cells were co-transfected with miR-127 promoter reporter, c-Jun (100 ng) and/or p53 plasmids (100 ng). *p<0.01, c-Jun or p53 *vs.* control; ^¥^p<0.01, c-Jun/p53 *vs.* p53. (*B-D*) Luciferase (Luc) activity was normalized by Renilla activity (act). (*E*) qPCR analysis of miR-127 and p53 expression in HepG2 cells transfected with control (con) or p53 siRNAs (si-p53) (20 nM), or in HCT116*p53^+/+^* (+) and HCT116*p53^−/−^* (-) cells. *p<0.01, si-p53 *vs.* control. (*F*) qPCR analysis of miR-127 expression in MHCC97H cells that were overexpressed with c-Jun and/or p53. *p<0.01, p53 *vs.* control (-); ^¥^p<0.01, c-Jun/p53 *vs.* p53.

Treatment of MHCC97H cells with TGFβ at the dose of 5 ng/ml was sufficient to cause a maximal induction of MMP13 mRNA (Figure 4A, left). Interestingly, TGFβ induced c-Jun mRNA (Fig. 4A, middle) but inhibited miR-127 levels (Fig. 4A, right) in a dose-dependent fashion. The results suggest that TGFβ is likely to repress miR-127 through c-Jun.

Indeed, transient transfection assays showed that overexpression of c-Jun, but not c-Fos, dose-dependently inhibited miR-127 promoter activity (Fig. 4B), and the inhibition of c-Jun was further enhanced by TGFβ (Fig. 4C, left). In contrast, TGFβ augmented c-Jun activation of the AP1 promoter, which served as a positive control (Fig. 4C, right). On the other hand, overexpression of miR-127 did not affect TGFβ activation of c-Jun (not shown).

Overexpression of p53 induced miR-127 promoter transactivation, which was antagonized by c-Jun (Fig. 4D), suggesting a direct activation of miR-127 transcription by p53. Our recent studies showed that p53 was abundantly expressed in HepG2 cells [15], [24]. Therefore, HepG2 cells were used to examine the effect of transient p53 knockdown on miR-127 expression. The levels of miR-127 were significantly reduced in HepG2 cells transfected with p53-siRNA (Fig. 4E, left and middle). The levels of miR-127 were also markedly lower in p53 mutant HCT116*p53^−/−^* cells compared with HCT116*p53^+/+^* cells (Fig. 4E, right). In addition, overexpression of p53 (Fig. 4F, left) induced miR-127 expression in MHCC97H cells and the effect of p53 was blocked by c-Jun (Fig. 4F, right). Overall, the results suggest that p53 activation of miR-127 is a common but not a cell type specific phenomenon.

### TGFβ Represses miR-127 Expression Through c-Jun Mediated ERK and JNK Pathways

**Figure 5:**
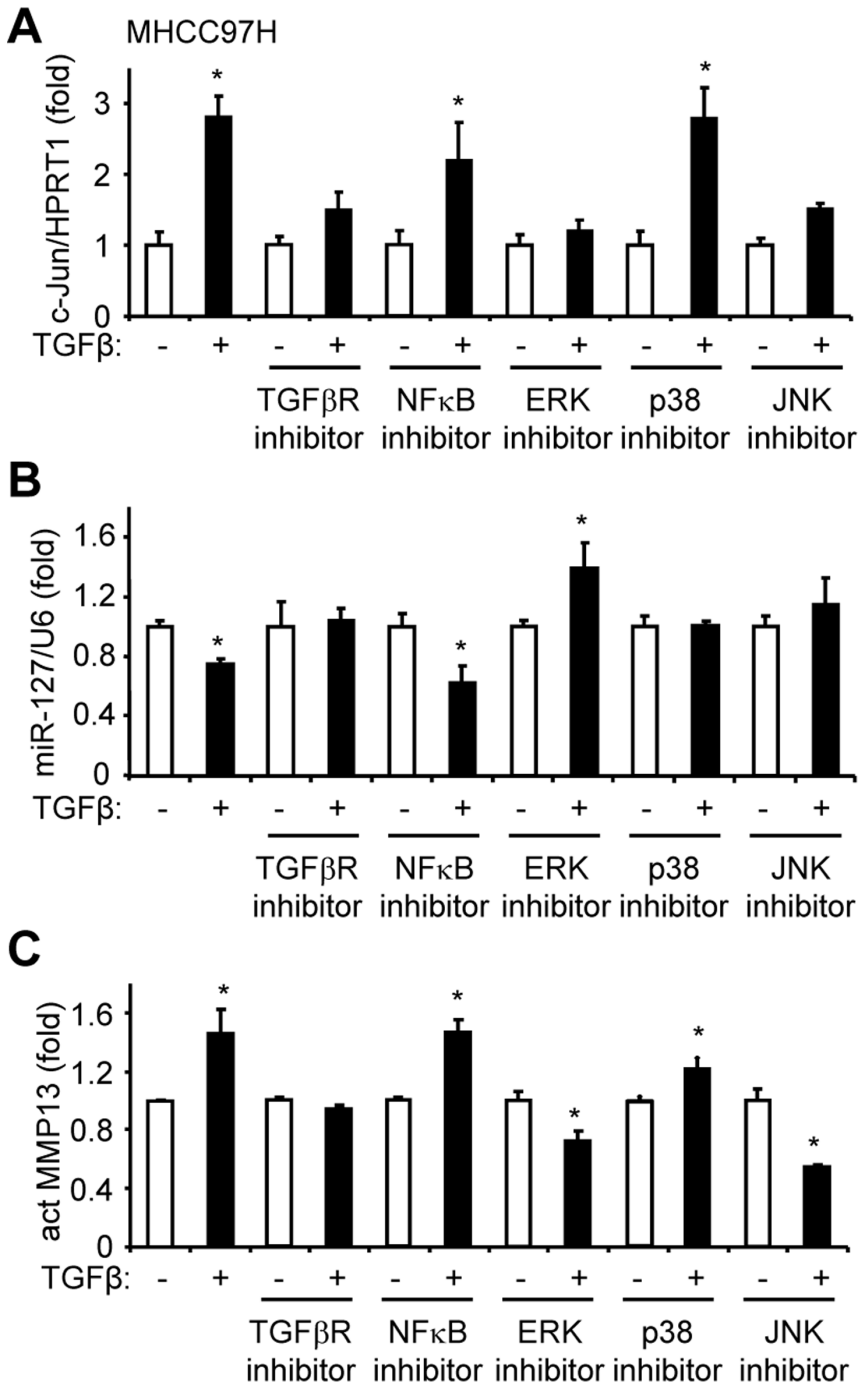
TGFβ enhances c-Jun-mediated repression of miR-127 via ERK and JNK pathways. (*A-C*) qPCR analysis of c-Jun (*A*), miR-127 (*B*), and MMP13 (*C*) mRNA expression in MHCC97H cells treated with TGFβR inhibitor (SB 431542, 10 µM), NFκB inhibitor (BAY-11-7082, 5 µM, ERK inhibitor (PD 98059, 50 µM), p38 inhibitor (SB 203580, 10 µM) and JNK inhibitor (SP 600125, 50 µM),) in the absence (−) or presence of TGFβ (+) (5 ng/ml). Statistical results represent the mean ± SD. *p<0.01, TGFβ (+) *vs.* TGFβ (−) group.

Because activation of TGFβ initiates multiple signaling pathways [25], [26], we sought to determine the specific pathways that are involved in the inhibition of miR-127 by c-Jun downstream of the TGFβ signaling. Inhibition of TGFβR, ERK, and JNK activation largely blocked TGFβ induction of c-Jun mRNA expression (Fig. 5A), whereas inhibition of NFkB and p38 mitogen-activated protein kinase (MAPKs) had no effect. Consistently, the repression of miR-127 by TGFβ was relieved by TGFβR, ERK, and JNK inhibitors, but not by an NFkB inhibitor (Fig. 5B). Interestingly, miR-127 repression by TGFβ was also blunted by the p38 MAPKs inhibitor, suggesting an alternate mechanism not involving c-Jun.

We also examined secreted MMP13 (active MMP13) protein in cell medium. Consistent with the mRNA results (Fig. 4A), TGFβ treatment induced act MMP13 (Fig. 5C). This induction was reversed by ERK and JNK inhibitors and diminished by a TGFβR inhibitor, but was not affected by p38 and NFkB inhibitors.

Overall, the results demonstrate that TGFβ represses miR-127 expression and activates MMP13 expression through c-Jun mediated ERK and JNK pathways, and NFkB signaling is not involved in such regulation.

### miR-127 is Downregulated in a Subset of HCC Specimens

**Figure 6:**
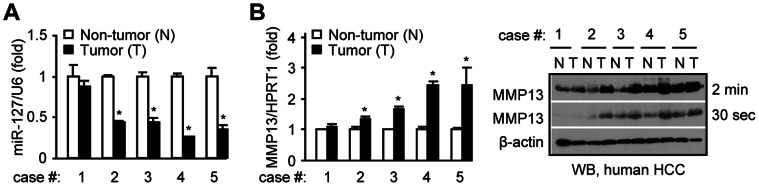
miR-127 is downregulated in a subset of HCC specimens. (*A-B*) qPCR analysis of miR-127 expression (*A*) and MMP13 mRNA (*B*, left), and Western blot (WB) analysis of MMP13 protein (*B*, right) in 5 pairs of surrounding controls and HCC specimens. *p<0.01, tumor *vs.* non-tumor.

We analyzed miR-127 expression in five HCC specimens (T) and compared it with matched normal surrounding livers (N) [27]. The miR-127 was downregulated in four out of five HCC (Fig. 6A), and the downregulation of miR-127 correlated negatively with the upregulation of MMP13 mRNA and protein (Fig. 6B).

## Discussion

The incidence and mortality rate of HCC have doubled in the US in the past four decades and are expected to double again over the next 10 to 20 years [28]. The incidence of HCC has shifted toward relatively younger ages, such that liver cancer is becoming a major health problem in the United States.

TGFβ figures prominently among gene signatures associated with HCC progression and metastasis [29]–[31], and tumors expressing TGFβ often display an invasive phenotype and high tumor recurrence [32]. Anti-TGFβ therapies are currently being developed and tested in pre-clinical studies [26], [33]. However, the TGFβ pathway is implicated in multiple homeostatic processes, which poses great challenges in TGFβ-targeted cancer therapy. More specific modulation of the signaling components downstream of TGFβ offers an attractive means of regulating the response of cancer cells to TGFβ signals.

**Figure 7:**
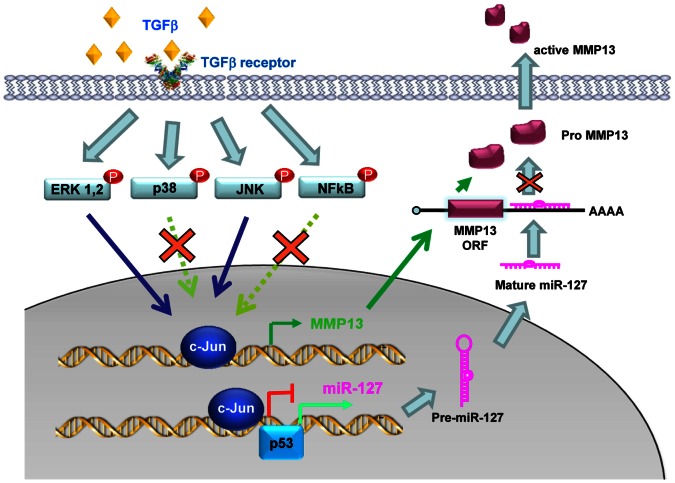
Model of negative-feedback regulation of miR-127 expression by TGFβ/c-Jun that controls MMP13 expression and stability in HCC. TGFβ activates the oncogene c-Jun through ERK and JNK pathways. The activation of c-Jun serves a dual function, which involves induction of MMP13 gene expression but repression of miR-127 gene transcription by inhibiting miR-127 promoter activity. c-Jun also antagonizes p53 activation of the miR-127 promoter and gene transcription. On the other hand, overexpression of miR-127 decreases MMP13 protein levels by binding to its 3′UTR and causing MMP13 degradation, thus diminishing TGFβ-mediated HCC migration.

Although downregulation of miR-127 was observed in rat liver during hepatocarcinogenesis [10], the functional role of miR-127 in HCC has remained elusive until now. Several findings are reported in this study (Fig. 7). First, we defined a role of miR-127 in inhibiting HCC migration. Second, we elucidated the underlying molecular basis by identifying MMP13, a gene downstream of the TGFβ signaling that is important in tumor invasion and metastasis, as a target for miR-127 inhibition. Third, we identified c-Jun as a transcriptional repressor and p53 an activator of miR-127 expression. Fourth, we showed that ERK and JNK pathways are involved in TGFβ-induction of c-Jun to activate the expression of MMP13 and repress miR-127. Fifth, we demonstrated that miR-127 is downregulated in a subset of HCC specimens that have active MMP13. Overall, our studies establish a novel feedback regulatory network between miR-127 and the TGFβ signaling, whereby miR-127 constrains TGFβ activation of HCC cell migration via inhibition of MMP13 function, and TGFβ represses miR-127 expression via activation of c-Jun. It should be noted that other unidentified miR-127 targets may also be involved in miR-127 regulation of HCC cell migration and invasion, which is currently under investigation.

Interestingly, induction of c-Jun by TGFβ is mediated by both ERK and JNK pathways, which is in agreement with the observation in human melanoma in which the rewired ERK signaling pathway upregulates JNK and activates c-Jun [34]. On the other hand, p38 protein kinases, another downstream mediator of the mitogen-activated protein kinase pathways [35], is not involved in TGFβ activation of c-Jun, but is involved in TGFβ repression of miR-127. This observation suggests that TGFβ can feedback repress miR-127 expression through both c-Jun-dependent and independent mechanisms. It would be interesting to determine in future studies whether miR-127 may play a role in TGFβ-mediated Smad activity.

It is noteworthy that miR-127 expression is downregulated in a subset of HCC specimens we analyzed. This is not unexpected, considering the fact that HCC is a heterogeneous disease caused by many agents such as viral and chemical carcinogenes or conditions such a chronic hepatitis and cirrhosis. Importantly, the TGFβ signature only occurred in a subset of HCC displaying invasive phenotype [29], [30] and the downregulation of miR-127 correlated with the activation of MMP13/TGFβ signaling. The results further support MMP13 as a target of miR-127, and also establish the functionality of miR-127 and MMP13 in the development of HCC.

MHCC97H cells display higher metastatic properties, and a stem cell population isolated from MHCC97H was used as an *in vivo* HCC metastatic model [28]. However, in our pilot studies using the entire MHCC97H cell population in an Orthotopic transplanted model, we did not observe lung metastasis, which differs from the report using the stem cell population [28]. This has limited our ability to test the *in vivo* effect of miR-127 in the inhibition of HCC metastasis. Nonetheless, our present studies establish for the first time a role of miR-127 in inhibiting HCC invasion *in vitro* and HCC growth *in vivo*. Future studies will be focused on using miR-127 knockout mouse model to further examine the function of miR-127 in HCC development and the use of this miR as a potential agent to diminish the amplified TGFβ signaling and modulate the invasiveness of HCC cells.
